# Supplementary material for: Sliding friction over individual aromatic bonds correlates with bond order
Source: Nat Commun. 2026 Apr 22;17:3694. doi: 10.1038/s41467-026-72128-x (PMC13103326; doi:10.1038/s41467-026-72128-x)
Supplement: Supplementary file 1 — Supplementary Information [file 41467_2026_72128_MOESM1_ESM.pdf]

Supplementary Information for

Sliding friction over individual aromatic bonds correlates with bond order

Shinjae Nam[1,2†], Lukas Hörmann[3,4†], Oliver Gretz[1†], Oliver T. Hofmann[5], Franz J. Giessibl[1,6], Alfred J. Weymouth[1,6*]

Affiliations (plus present addresses):

[1]Faculty of Physics, University of Regensburg; Regensburg, 93053, Germany.

[2]Center for Quantum Nanoscience, Institute for Basic Science (IBS), Seoul, 03760, South Korea.

[3]Department of Chemistry, University of Warwick; Coventry, CV4 7AL, UK

[4]Faculty of Physics, University of Vienna; Vienna, 1090, Austria

[5]Institute of Solid State Physics, Graz University of Technology; NAWI Graz, Graz, 8010, Austria.

[6]Regensburg Center for Ultrafast Nanoscopy (RUN), University of Regensburg; Regensburg, 93053, Germany.

*Corresponding author. Email: jay.weymouth@ur.de

† These authors contributed equally to this work

This PDF file includes:
Supplementary Figure 1-17
Supplementary Table 1
Supplementary Methods
Experimental
Simulation
Supplementary References

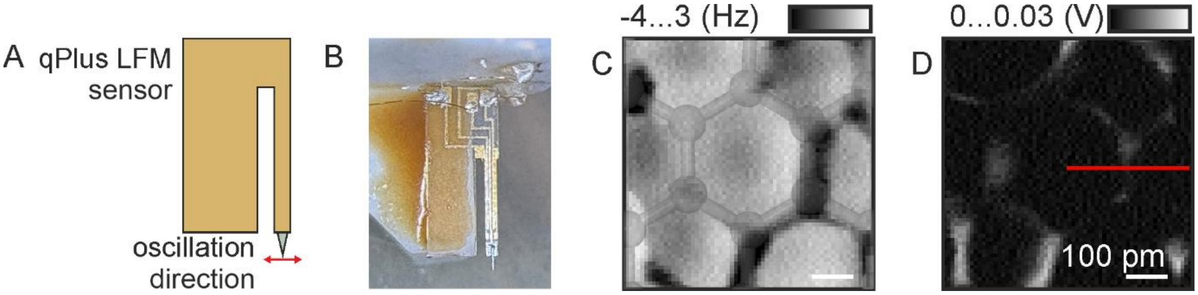

Supplementary Figure 1 | Lateral force microscopy. (A) Schematic of the LFM sensor (B) Photograph of the sensor. The qPlus quartz oscillator is mounted so that the tip oscillates laterally. (C) LFM frequency shift (Δf) and (D) raw drive signal A'_{drive} of Fig. 1c.

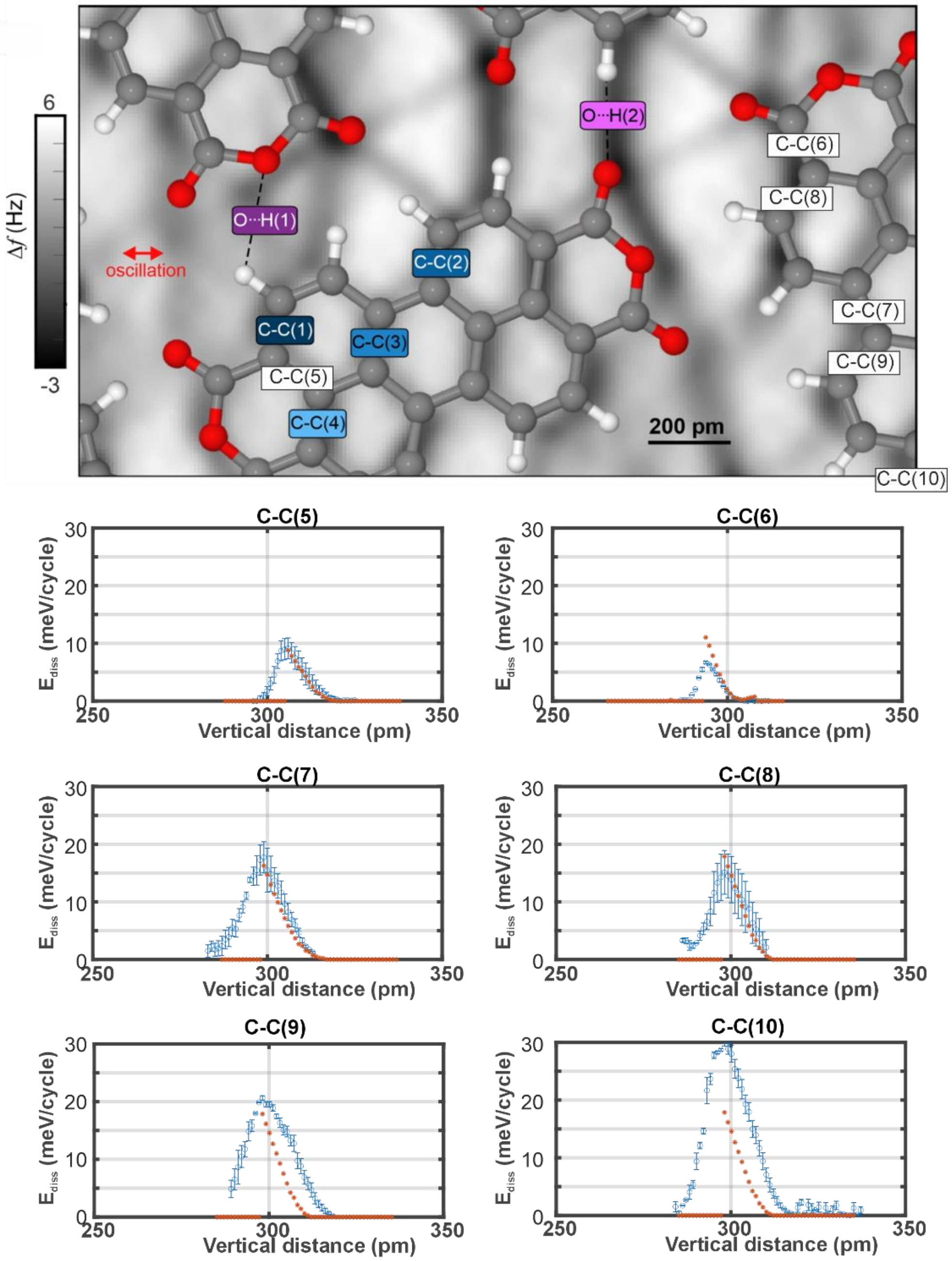

Supplementary Figure 2 | Energy dissipation over covalent bonds with different angles to the oscillation direction. Data are shown in blue with uncertainty, and simulated data are shown in orange. The data was acquired using the same CO tip and repeated 12 times. Error bars represent the standard deviation (SD) of 12 technical replicates. Data are presented as mean values ± SD (n = 12), where measurements were repeated at the same spatial location using the same CO-functionalized tip. Source data are provided in the Source Data file.

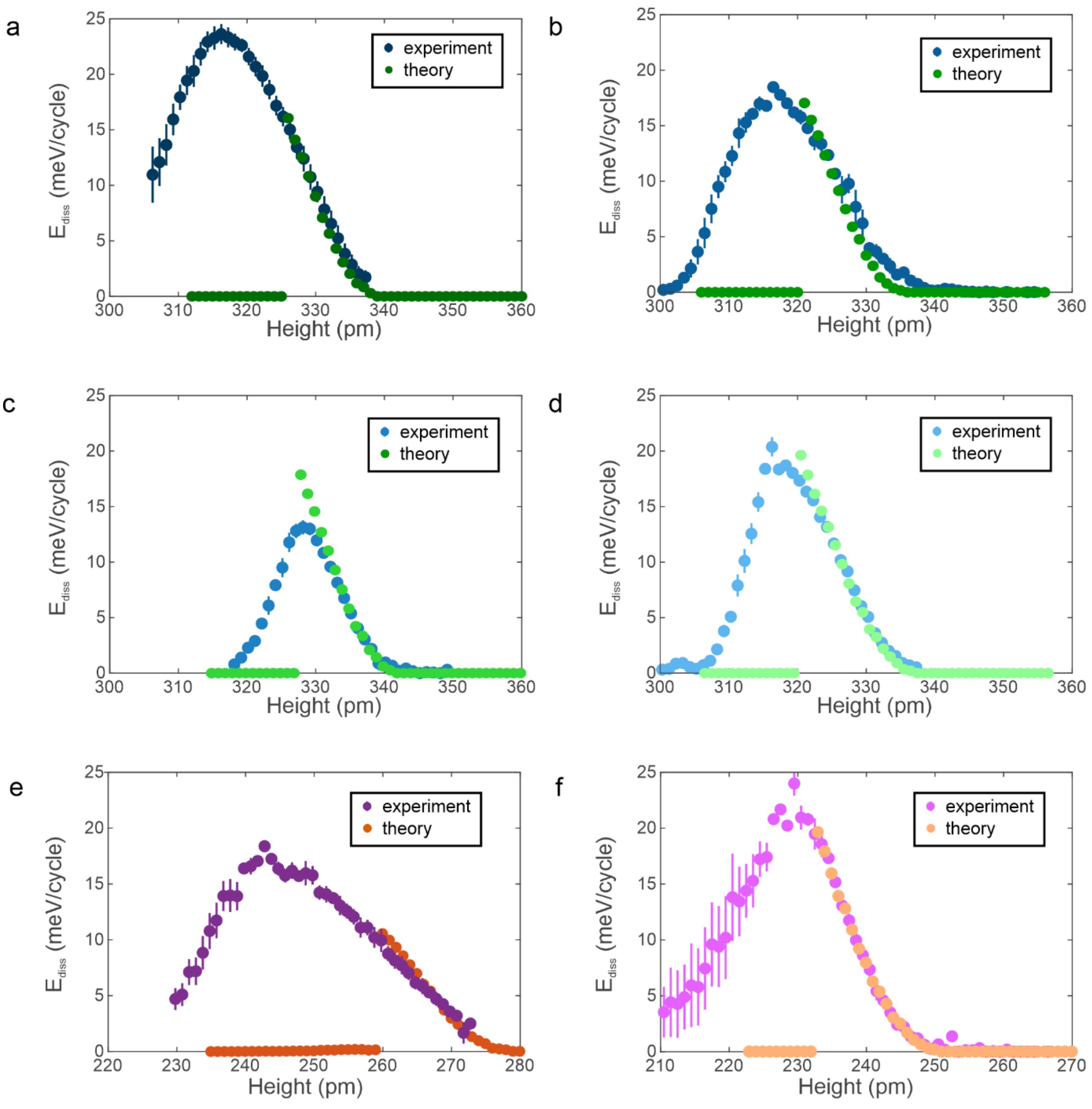

Supplementary Figure 3 | Theory and experimental curves in Figure 2 compared. The experiment z-axis is shifted so the increase in E_{diss} (as z decreases) is aligned with theory. The data was acquired using the same CO tip and repeated 12 times. Error bars represent the standard deviation (SD) of 12 technical replicates. Data are presented as mean values ± SD (n = 12), where measurements were repeated at the same spatial location using the same CO-functionalized tip. The labelling follows Fig. 2: a) C-C(1) b) C-C(2) c) C-C(3) d) C-C(4) e) O···H(1) f) O···H(2)

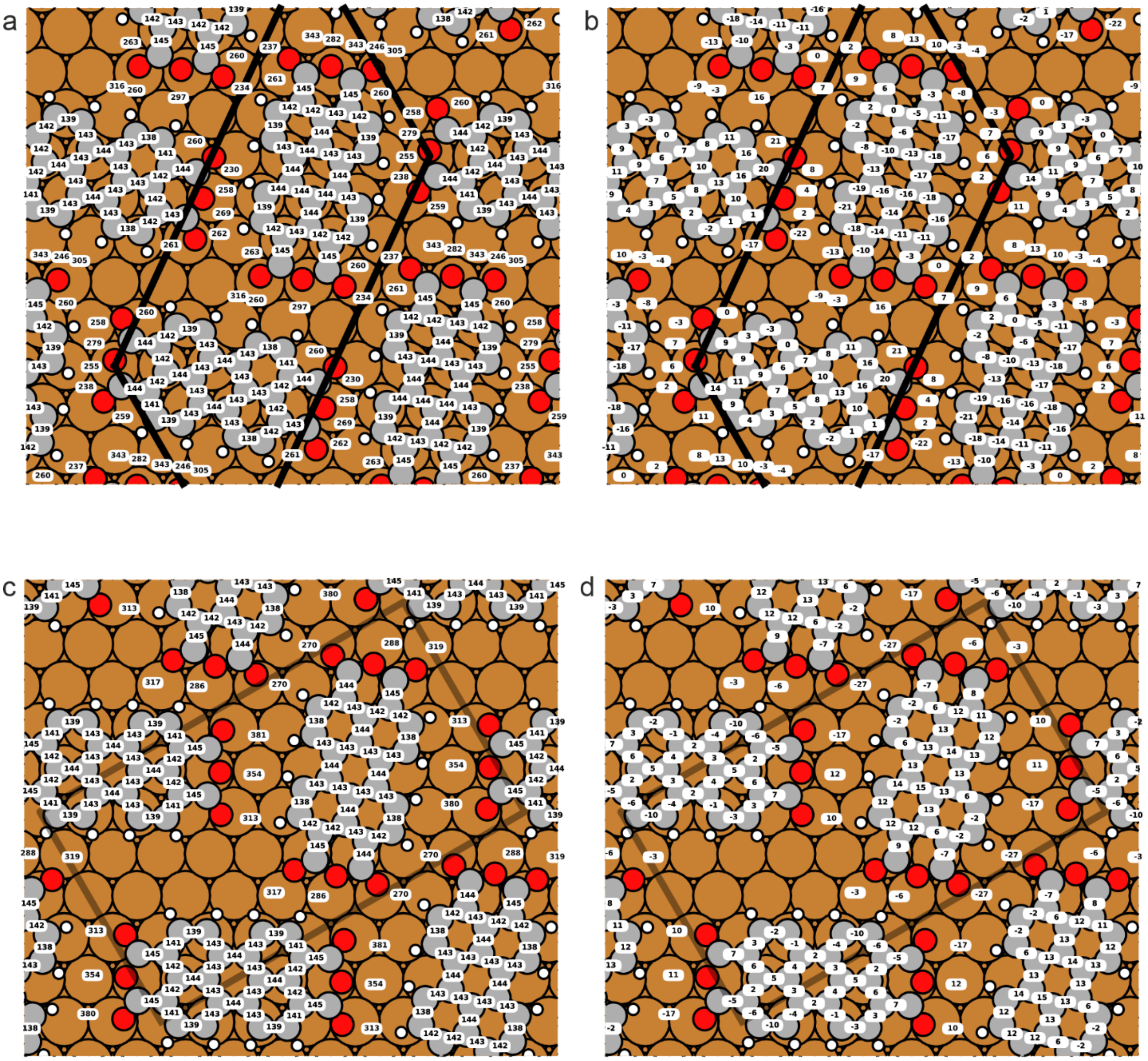

Supplementary Figure 4 | Relaxed position as a function of unit cell. Shown are the DFT-calculated relaxed bond lengths (a, c) and bond heights (b, d) of two different adsorption geometries of two PTCDA molecules above the Cu(111) surface. The two different unit cells are shown as black parallelograms in a, b, and gray rectangles in c, d. While there are small changes in bond lengths, there are substantial changes in the relative bond heights. The reference height of the bond heights is the average height of the PTCDA molecules.

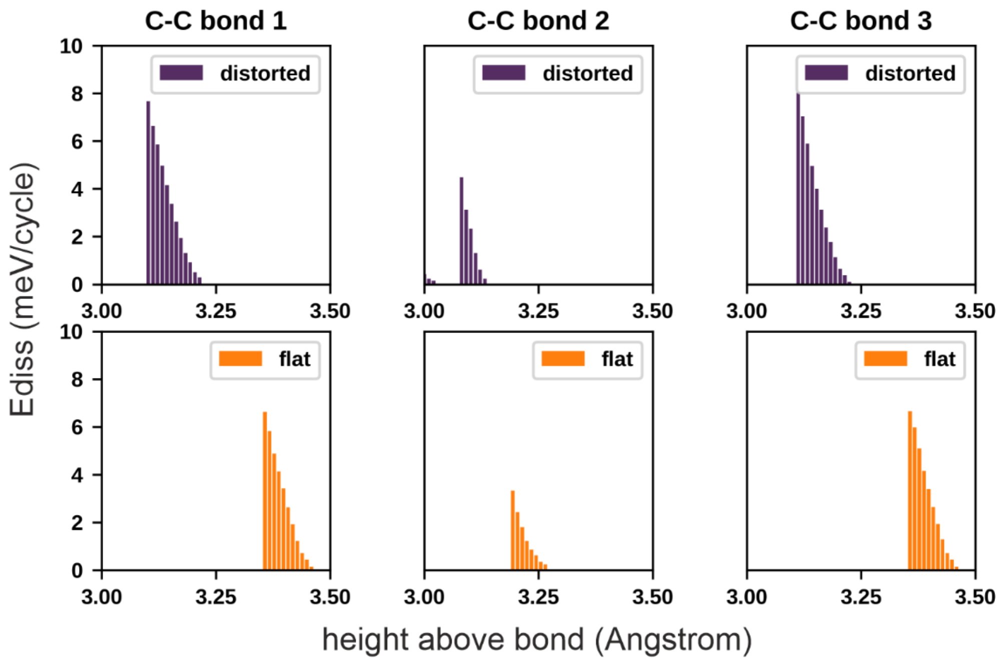

Supplementary Figure 5 | Sliding friction over different PTCDA geometries. The energy dissipation was calculated over several bonds using Lennard-Jones potentials for PTCDA in a flat (gas-phase) geometry, and in the distorted geometry when adsorbed as shown in Supplementary Fig. 4c and d. Distorted bonds tend to exhibit larger amounts of energy dissipation than flat bonds. Source data are provided in the Source Data file.

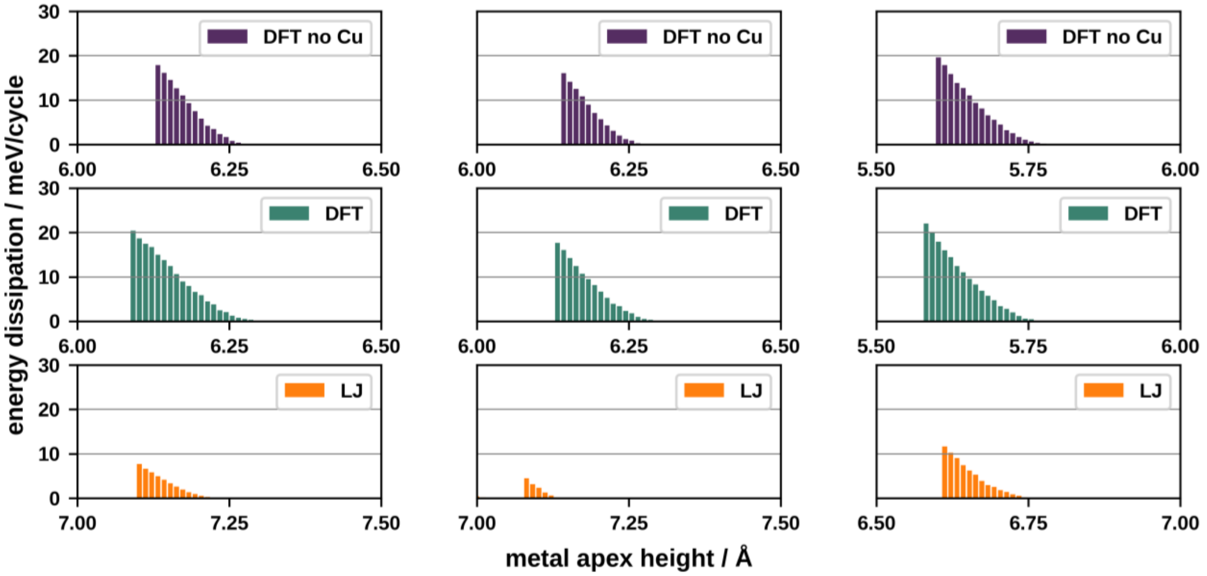

Supplementary Figure 6 | Comparison of energy dissipation calculations using (first row) DFT without a metal substrate, (middle row) DFT with substrate, and (bottom row) a commonly used Lennard Jones potential. First column: CC(1), second column: CC(3), third column: O···H(2). Comparing the first two rows, leaving out the substrate results in a small, constant decrease in energy dissipation. This is a result of attractive, non-site-specific vdW-interactions between the CO-molecule and the substrate, which lead to a lower onset of snapping and thus a slightly larger amount of energy dissipation. Comparing the top rows to the bottom row, the Lennard Jones potential yields energy dissipation prediction that significantly differs from the DFT results. We attribute this to the more rigorous treatment of the quantum-mechanical interactions between the CO-tip and the PTCDA molecules by DFT. Moreover, this demonstrates the necessity of using accurate first-principles methods if one wishes to determine quantitative predictions of LFM measurements. Source data are provided in the Source Data file.

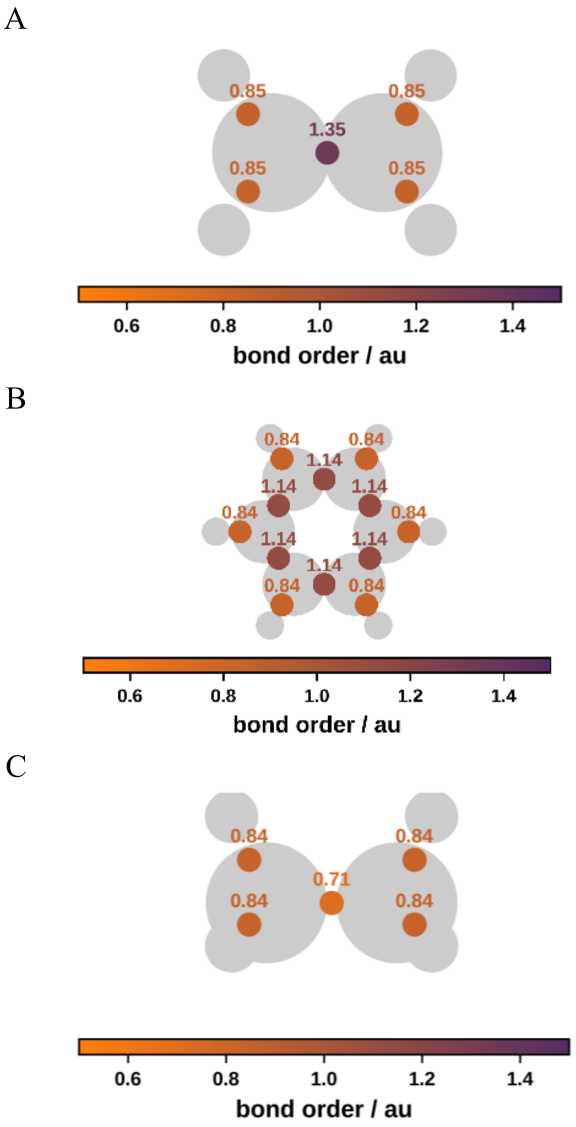

Supplementary Figure 7 | Mulliken bond order for (A) C_2H_4, (B) C_6H_6, and (C) C_2H_6.

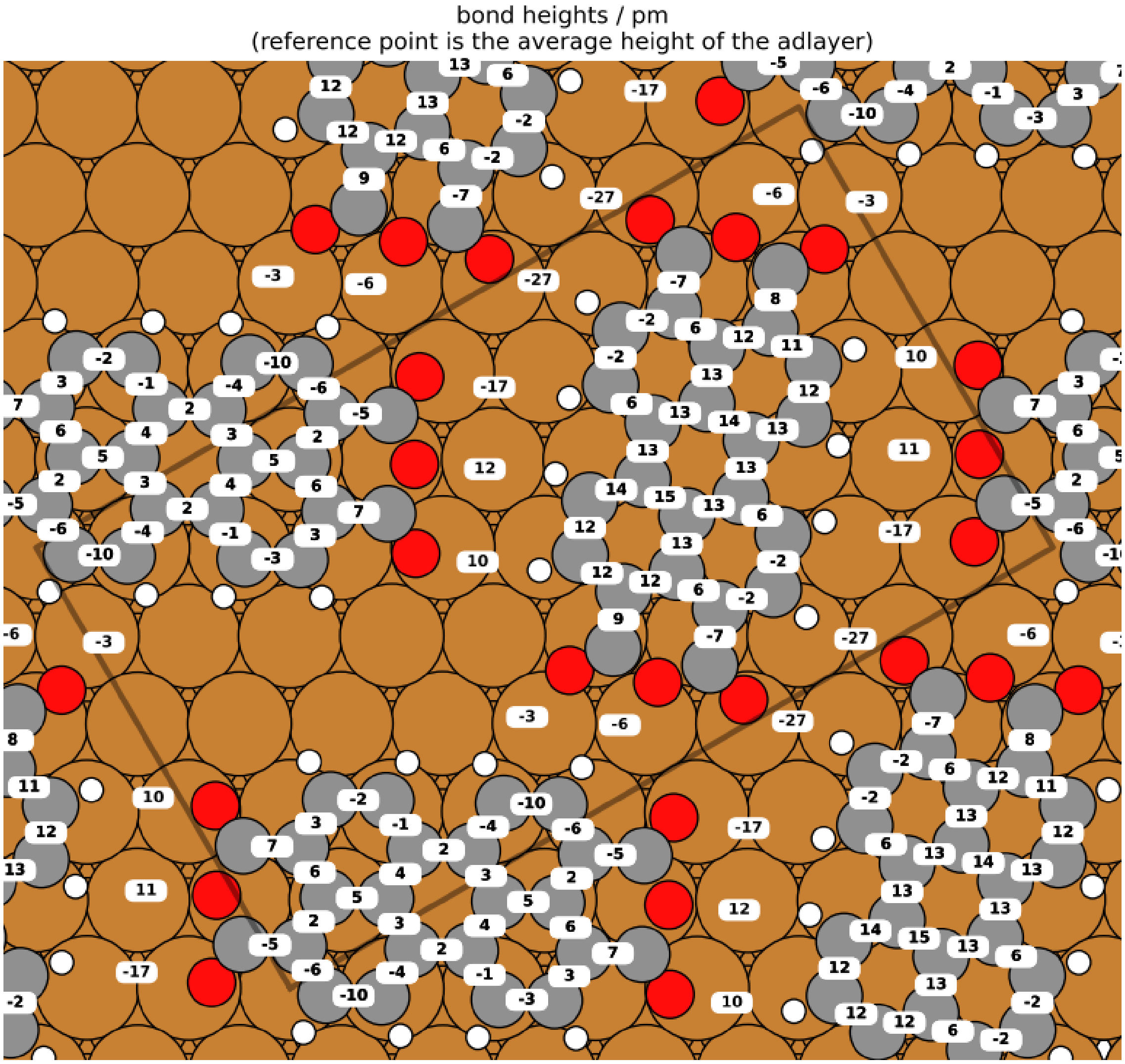

Supplementary Figure 8 | Height of bonds (picometer) with respect to the adlayer. The bond height is defined as the average height of the two atoms participating in the bond above the average height of all C, O and H atoms.

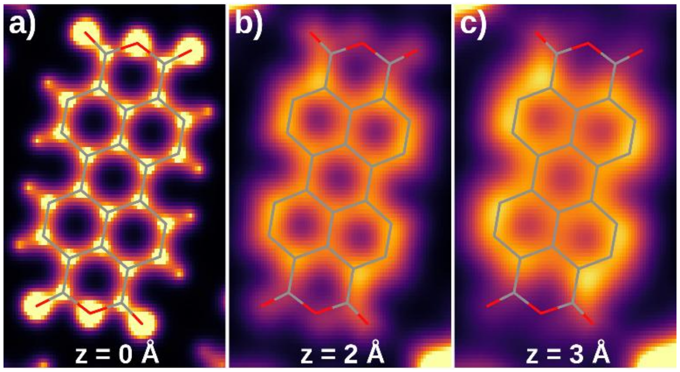

Supplementary Figure 9 | Electron density at various planes above the molecular plane. a) Electron density at the plane of the PTCDA. b) Electron density 200 pm above the molecular plane. c) Electron density 300 pm above.

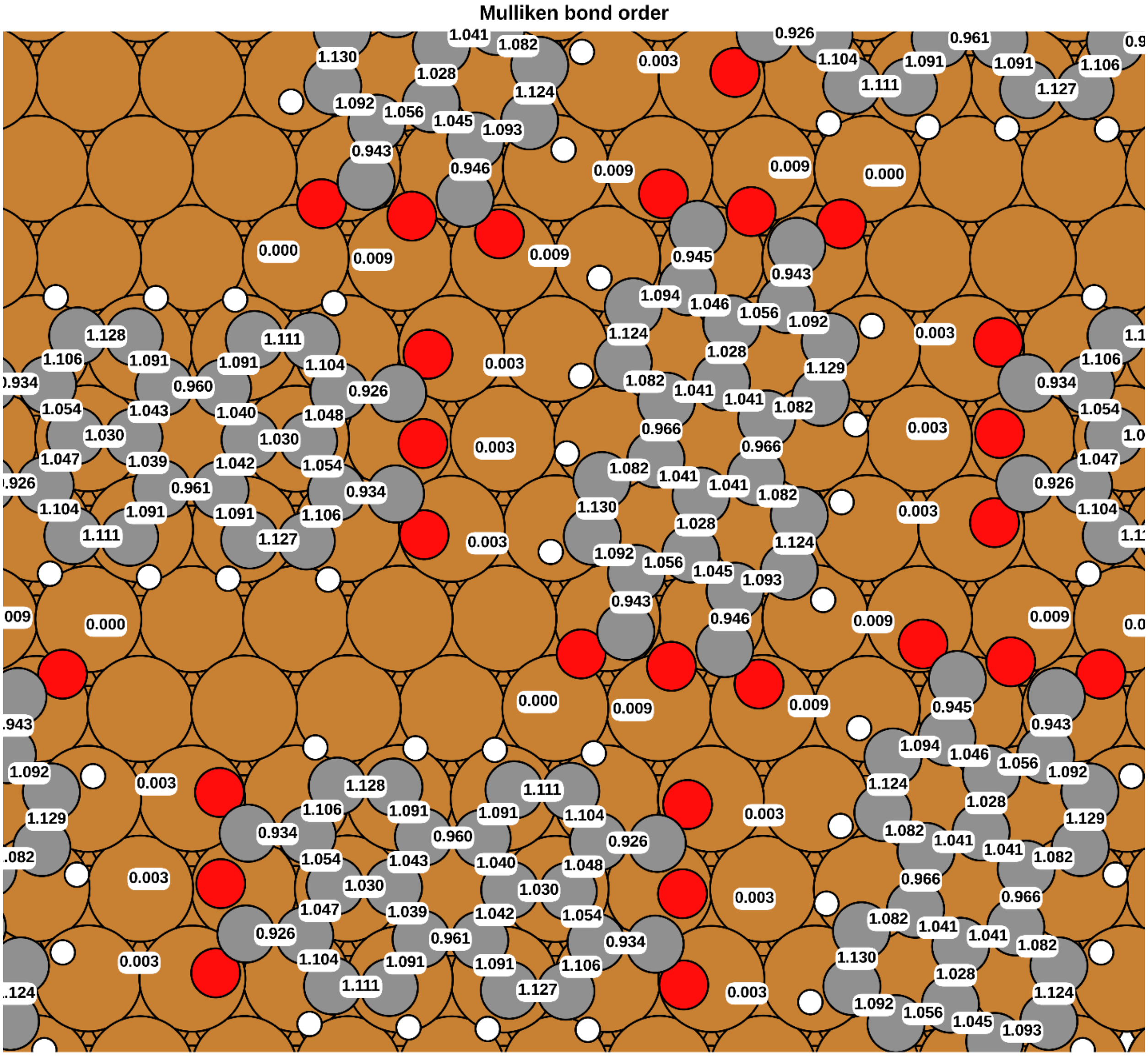

Supplementary Figure 10 | Mulliken bond order of all calculated bonds.

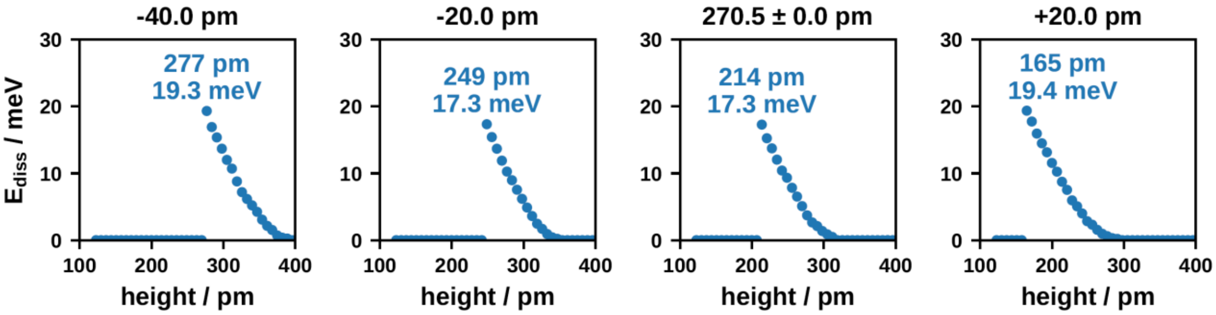

Supplementary Figure 11 | Dependence of the dissipated energy over an OH bond when artificially moving the OH atoms closer (-40 pm, -20 pm) than the calculated length (270.5 pm) and further (+20 pm). Note that the maximum energy dissipation is not a monotonic function of the distance. Source data are provided in the Source Data file.

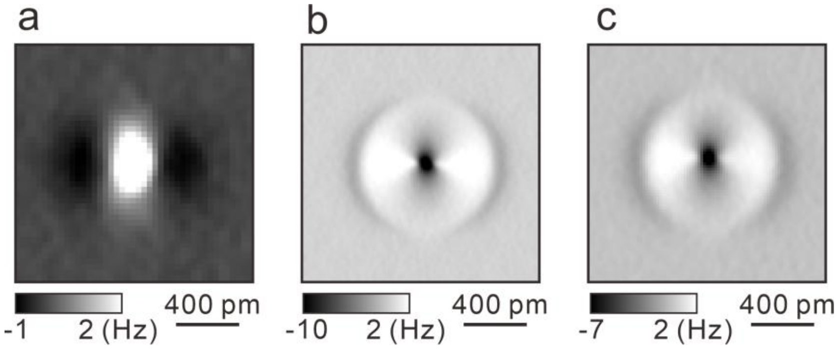

Supplementary Figure 12 | Characterization of the tip via a surface CO molecule. (a) CO imaged with a symmetric single-atom metal tip before CO pickup. (b) CO imaged with a CO-terminated tip prior to friction data acquisition. The tip termination is vital for reproducible images, therefore if the tip termination changes during scanning, the image contrast will change and it will be clear that the CO is no longer present. (c) CO imaged with the same CO-terminated tip after data acquisition, confirming stable tip termination throughout the measurement. Here, (b) was acquired before and after data shown in Figure 2 and (c) was acquired after.

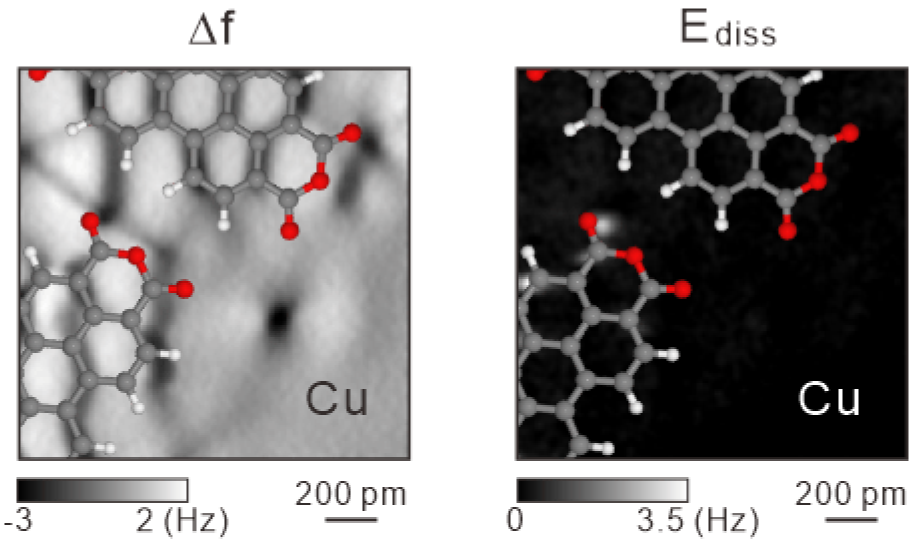

Supplementary Figure 13 | Excitation signal beyond the PTCDA islands. At heights where excitation can be observed over the PTCDA molecule, no excitation is observed beyond the edge of the molecular island. This is because the substrate does not directly contribute to the dissipation signal. The dark depression seen in the Δf image is a CO molecule and although this sits on the surface, there is no excitation signal observed over it.

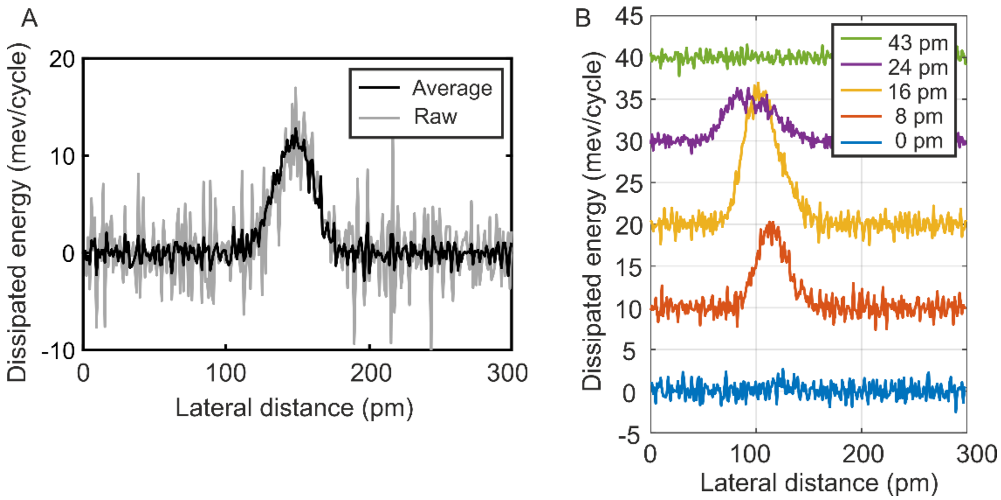

Supplementary Figure 14 | Analysis of E_{diss} data acquired over chemical bonds. (A) Raw data of E_{diss} (gray line) recorded above the red line shown in Supplementary Figure 1(D). Data were acquired as two lines along both the backward and forward scanning directions. These four lines of data were then averaged. Lines of E_{diss} were measured with a tip height difference of 1 pm. The lines as a function of height produce a 2D image which was then filtered with a Gaussian filter with a standard deviation of 0.5 pixels (black line). (B) Corresponding E_{diss} data plotted as a function of lateral (x) for various vertical (z) tip positions as a waterfall plot. The tip height (z) is relative, with z = 0 pm at the point of closest tip-sample distance. In the main text, the maximum values of E_{diss} from line scans in (B) were plotted as a function of tip height to compare energy dissipation across bonds. E_{diss} data were measured with 1 pm height intervals, averaged over multiple datasets for accuracy.

A

B

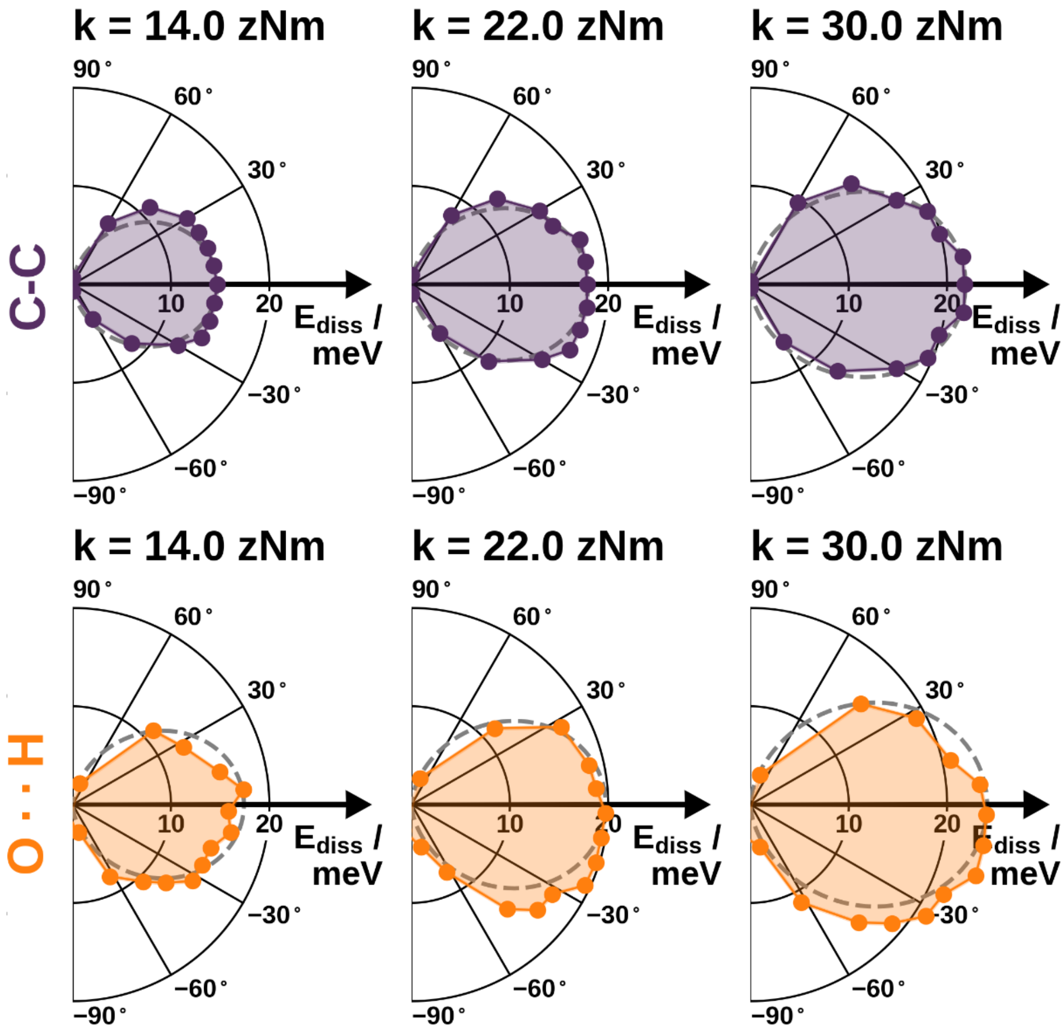

Supplementary Figure 15 | Energy dissipation is plotted as a function of the oscillation direction. An angle of 0° means that the oscillation direction is perfectly perpendicular to the bond. (A) Data for tips of various stiffness (torsional stiffness of the CO) over a covalent bond and (B) over a hydrogen bond. For angles ±10°, the dissipation does not strongly change. Source data are provided in the Source Data file.

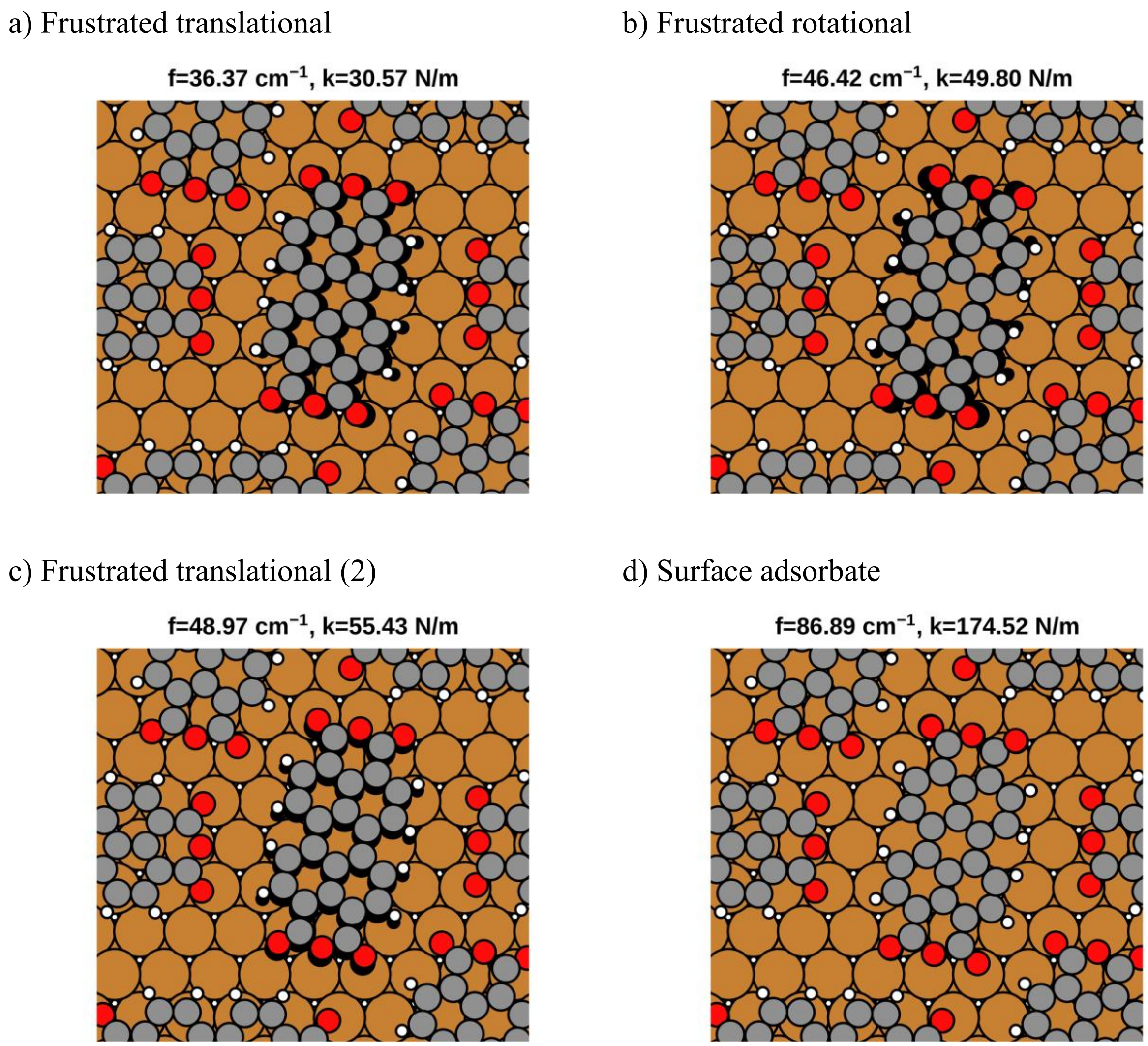

Supplementary Figure 16 | Four lowest vibration modes of the PTCDA molecule on a Cu(111) surface. The vibrational eigenvalues and eigenvectors for the PTCDA molecule in a close-packed adsorbate layer on the Cu(111) were determined using DFT. The central molecule is displaced using the eigenvector of the respective vibration mode. The original position is indicated by the black shadow below the central molecule. A) Frustrated translational mode b) Frustrated rotational mode c) Higher frustrated translational mode (labelled 2) d) Surface adsorbate mode.

Vibration mode	Vibration frequency	Lateral spring constant
Frustrated translation	36 cm^{-1}	31 N/m
Frustrated rotation	46 cm^{-1}	50 N/m
Frustrated translation (2)	49 cm^{-1}	55 N/m
Surface adsorbate	87 cm^{-1}	175 N/m

Supplementary Table 1 | Softest vibration modes of PTCDA molecule on Cu(111). The lateral spring constants of these modes are at least two orders of magnitude larger than the lateral spring constant of the CO-tip which has been determined to be 0.24 N/m(*14*). Therefore, it is safe to assume that the lateral motion of the CO-tip has no influence on the substrate that is being probed.

Supplementary Methods

Experimental:

In frequency-modulation LFM, the amplitude of oscillation is set, and the drive signal, A'_{drive} required to maintain this amplitude is recorded. A'_{drive} is the signal sent to the excitation required to maintain the amplitude and is recorded in units of V. If the drive signal increases, it indicates that there is energy loss during the tip's oscillation, referred to as energy dissipation. This is then converted to energy dissipation, E_{diss}, with the unit of meV per cycle(*1*):

$$E_{diss} = \frac{\pi k A^2}{Q}\left(\frac{A'_{drive}}{A_{drive}} - 1\right) \quad (1)$$

Here k is the sensor stiffness, A is the oscillation amplitude, and Q is the quality factor of the sensor. A_{drive} is the drive signal when the tip is far from the surface, including only a contribution from the internal dissipation of the sensor (Q). E_{diss} represents the work done on the cantilever during one oscillation cycle.

E_{diss} is recorded simultaneously as the frequency shift (Δf, unit of Hz), which reflects the conservative interaction between the tip and the sample. The Δf image corresponding to the data shown in Fig. 1c is shown in Supplementary Fig. 14(C). With the Δf image, the centre of the chemical bond can be clearly identified.

Simulation

In essence there are three components to simulating energy dissipation:

1) Relax the PTCDA on the surface (DFT-based determination of the adlayer structure)
2) Describe the potential energy surface that the CO encounters above the surface using a machine-learning algorithm trained on DFT data (Interaction of the CO molecule with the surface)
3) Simulate an oscillation of the tip with the above potential energy landscape and considering the tip as a torsional spring (Calculating dissipation)

These steps are described below in more detail.

DFT-based determination of the adlayer structure

First, the atomic positions of two PTCDA molecules above the Cu(111) surface were determined. Calculations were done using the FHI-aims quantum chemistry code.(*2*) We used the PBE exchange-correlation functional(*3*) and the TS^{surf} van der Waals correction scheme(*4*, *5*). The default tight species settings were employed together with a generalized Monkhorst-Pack k-grid(*6*) with 44 k-points. Furthermore, we use the repeated slab approach, where we approximate the substrate using a slab with 5 layers, which are electrostatically decoupled using the dipole correction(*7*). To perform geometry optimizations, all atoms are relaxed.

The commensurate unit cell for the DFT modelling was taken from Wagner et al.(*8*). Two PTCDA molecules were placed in the unit cell using the geometry suggested by the experiment. To attain the global minimum structure, we perform a geometry optimisation based on this experimental starting geometry. Thereby the molecule as well as the first two layers of the copper substrate were allowed to relax, until the remaining force falls below 0.01 eV/Å on each atom.

Interaction of the CO molecule with the surface

The potential energy surfaces (PES) were calculated to describe the interaction of a CO-molecule with the sample surface. For this purpose, we use a machine-learning model based on

Gaussian process regression and radial distance functions, which we have previously employed to describe PESs of organic/inorganic interface systems.(*9*) The training data for these potentials is determined using DFT using the same settings as explained above. Separate PESs were determined for each probed bond. Example PESs are shown in Figs. 4e and f. Between 250 and 300 data points were used to train a PES for a given bond. The training data was chosen such that we reached a prediction accuracy (based on a leave-one-out-cross-validation-error root-mean-square-error) of within 2 meV for interaction between the CO-molecule and the sample surface. Supplementary figure 17 shows a learning curve for the C-C(1) bond.

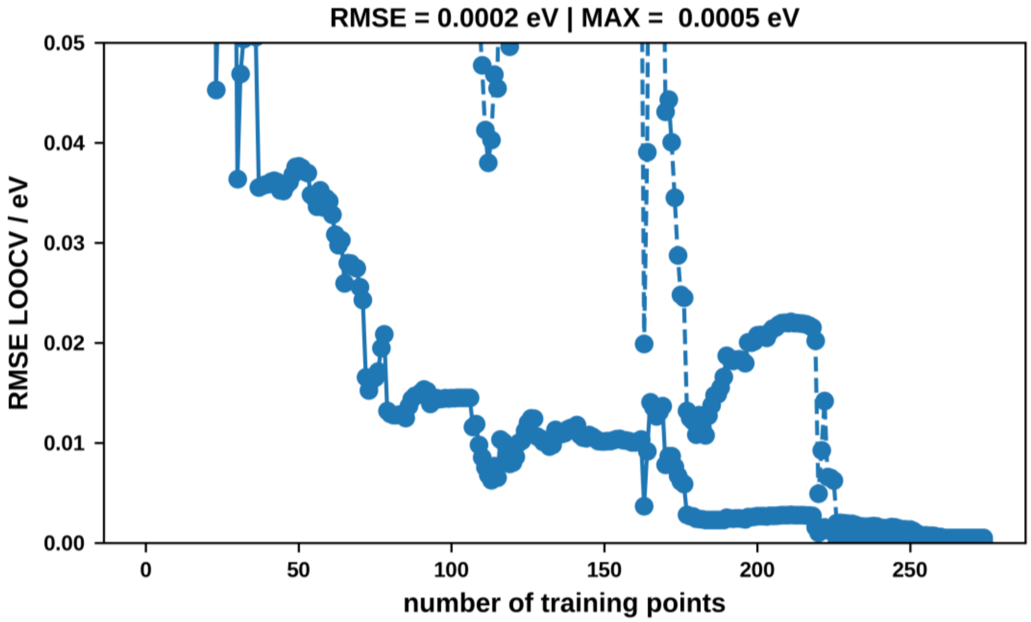

Supplementary Figure 17 | Learning curve for C-C(1) bond The leave-one-out-cross-validation-error root-mean-square-error (LOOCV RMSE, solid line) and the maximimum prediction error (dashed line) are shown.

Calculating dissipation

The snapping model is an extension of the one we described previously in Ref. (*10*). It is used to calculate both frequency shift and energy dissipation over an oscillation cycle assuming:

- The atoms of the surface are static
- The atoms of the tip are static except for the CO molecule at the apex
- The CO at the apex behaves like a torsional spring

The position of the apex metal atom of the tip is specified. Then the tip oscillates laterally from that point. It does so in space (i.e. not in time) and at each position finds the low-energy position (angle) of the CO at the tip apex based upon its previous position. That is, the CO can only relax to the local energy minimum, not the global energy minimum. In contrast to our previous model, the CO is allowed to relax in two directions.

Within each oscillation, the metal tip apex is moved laterally to the next point according to the formula:

$$x(t) = x_0 + A\cos(2\pi f_0 t) \quad (2)$$

Where x_0 is the average lateral position of the metal tip apex in the oscillation direction, A is the oscillation amplitude, and f_0 is the centre frequency of the sensor.

At each step, the CO-tip assumes an equilibrium deflection angle. This position is the local low energy position based upon its previous position, the interaction with the surface, and the torsional spring constant. Then the force resulting only from the interaction of the tip with the surface (and not for the torsional spring) is calculated(*11*) via the potential energy surface described above. The energy dissipation is then calculated by determining the convolution of the force $F(t)$ acting on the CO-tip with the phase of the oscillation, for example in Ref. (*12*).

$$E_{diss} = 2\pi A f_0 \int_0^{1/f_0} F \sin(2\pi f_0 t)\ dt \tag{3}$$

Determining bond order

We determined the bond order using Mulliken population analysis based on the orbital populations calculated using the FHI-aims quantum chemistry code. The FHI-aims quantum chemistry code describes the electronic wave function using a linear combination of atom-centred orbitals based on spherical harmonics and radial function (the latter include cut-offs at large distances).(*13*) The wave function is given by a series expansion in the basis of the atom-centred basis functions:

$$\phi_i = \sum_r c_{ri}\, \chi_r \tag{4}$$

using the series expansion of the wave function, we can determine the total number of electrons by summing over the expansion coefficients c_{ri} and the overlap matrix S_{rs}:

$$N = \sum_i \sum_{r,s} 2\, c_{ri}^{*} c_{si} S_{rs} = \sum_{r,s} P_{rs}\, S_{rs} \tag{5}$$

where we have used $P_{rs} = \sum_i 2\, c_{ri}^{*} c_{si}$. By splitting the summation into sums over all basis function centred on atoms A and B, we can determine the bond order:

$$N = \sum_A \sum_{r,s \in A} P_{rs}\, S_{rs} + \sum_{A<B} 2 \sum_{r \in A} \sum_{s \in B} P_{rs}\, S_{rs} = \sum_A q_A + \sum_{A<B} q_{AB} \tag{6}$$

We can interpret the second contribution q_{AB} as the bond order of the bond between the atoms A and B.

To benchmark this method of bond order determination, we calculate bond orders for simple molecules using identical settings to the calculations presented in the manuscript. Specifically, we compare the C=C double bond in C_2H_4 to the C-C single bond in C_2H_6. The C=C double bond in C_2H_4 has a bond order of 1.35, while the C-C single bond in C_2H_6 has a bond order of 0.71.

Calculating the correlation and confidence

First we determine the Pearson correlation coefficient r between the bond order and the energy dissipation. For this we find a value of $r = 0.68$. This value indicates a moderate to strong positive linear relationship between the the bond order and the energy dissipation.

To test if the correlation coefficient is meaningful, we use a Student's t-test. Here we first calculate t-score:

$$t_{score} = r \cdot \sqrt{\frac{n-2}{1-r^2}} \tag{7}$$

Here n is the sample size and r is the Pearson correlation coefficient. To attain a percentage value for the confidence we evaluate the cumulative distribution function of the Student's t using t_{score}. We use a one-tailed test, since we are interested in testing the validity of a positive correlation coefficient. We find a significance of 99.8%. This high significance level means there is a very low probability (0.2%) that the observed correlation is due to random chance.

Supplementary References

1. F. J. Giessibl, Advances in atomic force microscopy. *Rev. Mod. Phys.* **75**, 949–983 (2003).
2. V. Blum, R. Gehrke, F. Hanke, P. Havu, V. Havu, X. Ren, K. Reuter, M. Scheffler, Ab initio molecular simulations with numeric atom-centered orbitals. *Comput. Phys. Commun.* **180**, 2175–2196 (2009).
3. J. P. Perdew, K. Burke, M. Ernzerhof, Generalized gradient approximation made simple. *Phys. Rev. Lett.* **77**, 3865–3868 (1996).
4. V. G. Ruiz, W. Liu, E. Zojer, M. Scheffler, A. Tkatchenko, Density-functional theory with screened van der Waals interactions for the modeling of hybrid inorganic-organic systems. *Phys. Rev. Lett.* **108**, 146103 (2012).
5. A. Tkatchenko, M. Scheffler, Accurate Molecular Van Der Waals Interactions from Ground-State Electron Density and Free-Atom Reference Data. *Phys. Rev. Lett.* **102**, 073005 (2009).
6. J. Moreno, J. M. Soler, Optimal meshes for integrals in real- and reciprocal-space unit cells. *Phys. Rev. B* **45**, 13891–13898 (1992).
7. J. Neugebauer, M. Scheffler, Adsorbate-substrate and adsorbate-adsorbate interactions of Na and K adlayers on Al(111). *Phys. Rev. B* **46**, 16067–16080 (1992).
8. T. Wagner, A. Bannani, C. Bobisch, H. Karacuban, R. Möller, The initial growth of PTCDA on Cu(111) studied by STM. *J. Phys. Condens. Matter* **19**, 056009 (2007).
9. L. Hörmann, A. Jeindl, O. T. Hofmann, From a bistable adsorbate to a switchable interface: tetrachloropyrazine on Pt(111). *Nanoscale* **14**, 5154–5162 (2022).
10. A. J. Weymouth, E. Riegel, O. Gretz, F. J. Giessibl, Strumming a Single Chemical Bond. *Phys. Rev. Lett.* **124**, 196101 (2020).
11. P. Hapala, G. Kichin, C. Wagner, F. S. Tautz, R. Temirov, P. Jelínek, Mechanism of high-resolution STM/AFM imaging with functionalized tips. *Phys. Rev. B* **90**, 085421 (2014).
12. M. Ondráček, P. Hapala, P. Jelínek, Charge-state dynamics in electrostatic force spectroscopy. *Nanotechnology* **27**, 274005 (2016).
13. V. Blum, R. Gehrke, F. Hanke, P. Havu, V. Havu, X. Ren, K. Reuter, M. Scheffler, Ab initio molecular simulations with numeric atom-centered orbitals. *Comput. Phys. Commun.* **180**, 2175–2196 (2009).
14. A. J. Weymouth, T. Hofmann, F. J. Giessibl, Quantifying Molecular Stiffness and Interaction with Lateral Force Microscopy. *Science.* **343**, 1120 (2014).
