# Supplementary material for: Sliding friction over individual aromatic bonds correlates with bond order
Source: Nat Commun. 2026 Apr 22;17:3694. doi: 10.1038/s41467-026-72128-x (PMC13103326; doi:10.1038/s41467-026-72128-x)
Supplement: Supplementary file 2 — Description of Additional Supplementary File [file 41467_2026_72128_MOESM2_ESM.pdf]

The Description of Additional Supplementary Files

Supplementary Movie 1: Conceptual model of energy dissipation at various tip heights.
